# One-step construction of carbon nanoparticle/graphene oxide nanofiltration membranes with uniform sandwich structure for enhanced water purification†

**DOI:** 10.1039/d5ra00454c

**Published:** 2025-03-28

**Authors:** Xue Zhang, Ziyi Sang, Leiyang Xue, Lianwen Zhu

**Affiliations:** a College of Chemistry and Materials Science, Zhejiang Normal University Jinhua 321004 Zhejiang China; b School of Biology and Chemical Engineering, Jiaxing University Jiaxing 314001 Zhejiang China lwzhu@zjxu.edu.cn

## Abstract

Graphene-based membranes have great potential for water purification. However, it is still a challenge to achieve high solute rejection at high water flow by controlling the water permeation channel. Herein, carbon nanoparticles (CNPs) were uniformly sandwiched between graphene oxide (GO) sheets by one-step vacuum-assisted filtration of CNPs and GO mixed solution, resulting in the formation of CNPs/GO composite nanofiltration membranes with uniform sandwich structure. The addition of CNPs in the composite membrane could help to form a continuous transverse channel of water permeation and greatly increase the water flow. The results showed that the CNPs/GO composite membrane with a mass ratio of 20% exhibited the best performance. The pure water flow rate was 49.9 L m^−2^ h^−1^, which was 21 times higher than that of the pure GO membrane. The rejection rate for four different organic dyes exceeded 97%. The rejection rate for methylene blue (MB) was still 94.7% after 8 recycling cycles. In addition, the membranes allow the penetration of salts, which makes them promising for dye wastewater desalination. This study provides a simple and effective strategy to tune the channel microstructure of the composite membranes and increases the understanding of the important role of the sandwich particles in achieving a better performance of the membranes.

## Abstract 2

Carbon nanospheres (CNPs) were uniformly inserted between adjacent graphene oxide (GO) nanosheets for the construction of GO/CNPs membranes with obvious improved water flux and high rejection rate.

## Graphical abstract

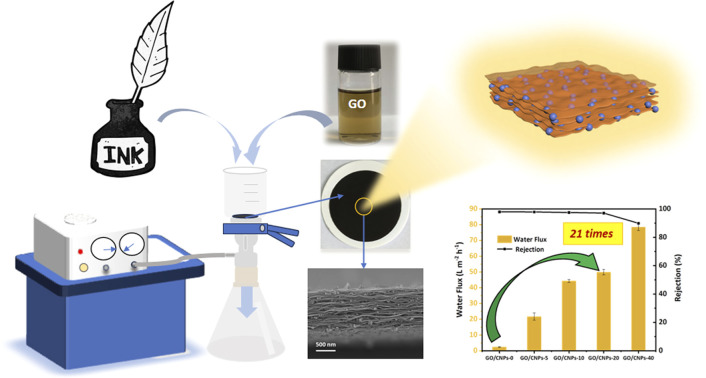

## 1 Introduction

Both the demand for fresh water and the volume of wastewater have increased exponentially with the world's growing population and rapid industrial development.^1,2^ The scarcity of clean water is one of the most pressing issues facing humanity today, especially in developing countries. By 2025, two-thirds of the world's population will face water scarcity, according to water resource management trends.^3^ To address this problem, several advanced technologies have been developed to remove aqueous contaminants for the production of clean water, including adsorption techniques,^4,5^ photocatalytic technology,^6,7^ and membrane separation methods.^8^ Among these, membrane separation, such as reverse osmosis and nanofiltration, is considered one of the most effective technologies due to its advantages in terms of ease of operation, low energy consumption, continuous production, no added chemicals and low environmental impact.^9,10^ The use of nanofiltration in water purification and dye separation has attracted increasing attention because it rejects organic molecules while allowing water molecules to pass through freely.^11^ However, most conventional nanofiltration membranes are made from polymer-based materials such as polyamide, polyvinyl alcohol and cellulose. Disadvantages such as poor chemical stability and thermal stability limit their use in the real world.^12–14^

Ultrathin two-dimensional (2D) materials,^15–19^ such as graphene and its derivative graphene oxide (GO), are emerging as building blocks for the development of high-performance membranes.^19^ Previous studies have shown that GO membranes have many advantages, including high solute rejection, excellent mechanical strength, good flexibility, and good chemical and thermal stability.^20,21^ As a result, many methods have been developed to prepare GO membranes. These include vacuum filtration^22^ spray method,^23^ layer-by-layer self-assembly method,^24^ and solvent casting method.^25^ Due to the strong π–π stacking interaction and hydrogen bonding, GO nanosheets are tightly stacked in a membrane, resulting in the formation of unique 2D water channels of 0.4–1.3 nm in size between two adjacent GO nanosheets.^26^ Therefore, GO membranes show tremendous potential in wastewater purification and seawater desalination. However, the water flow of pure GO membranes (1.7–21.4 L m^−2^ h^−1^) is much lower than that of conventional polymer membranes (50 L m^−2^ h^−1^) which hinders their widespread application.^27,28^ Previous studies have shown that the transport of water molecules in GO membranes mostly depends on the transverse channels between the GO sheets and the longitudinal pores between the edges of the GO sheets.^29^ Therefore, the key to achieving high performance GO membranes is to increase the interlayer spacing accordingly. This is because there is a trade-off between permeability and molecule rejection when the interlayer spacing is increased.

To overcome this problem, a variety of inorganic materials (*e.g.* ZnO,^30^ TiO_2_,^31^ SiO_2_,^22^ halloysite nanotubes,^32^ C_3_N_4_,^33^) have been incorporated into the adjacent GO nanosheets to adjust the membranes interlayer distance. For example, Long *et al.*^30^ fabricated ZnO/rGO membrane by growing ZnO on GO followed by a vacuum filtration process. By acting as rigid pillars, the ZnO nanoparticles not only increase the distance between the rGO sheets, but also create narrow tortuous paths between the 2D nanochannels for size-exclusion separation of dye molecules. Deng *et al.*^22^ introduced small-sized SiO_2_ nanoparticles into the GO membrane by using the vacuum filtration method, which resulted in the formation of continuous transverse channels in the composite membrane and greatly enhanced water flow rate (72.8 L m^−2^ h^−1^). Han *et al.*^34^ fabricated high performance GO-based composite membranes with remarkable water permeability and selectivity by using the combination of GO and unzipped CNTs. The obtained composite membranes exhibited better hydrophilicity and 10-fold higher water permeability compared with the pure GO membrane. In the case of sandwiched GO membranes, the dispersion and stability of the inorganic particles and the GO in aqueous solutions are very different, making it difficult for the inorganic particles to be uniformly inserted into the adjacent GO nanosheets. As a result, the construction of highly uniform sandwiched GO membranes using simple methods remains a major challenge.

In this paper, carbon nanospheres (CNPs) were uniformly placed between adjacent GO nanosheets to build CNPs sandwiched GO membranes by simple filtration of CNPs and GO mixture. The pure water flow, selectivity, antifouling performance and stability of the composite membrane were investigated. The results show that oxygen-containing groups such as hydroxyl and carbonyl groups on the surface of CNPs bind them firmly to GO nanosheets, resulting in an impressively uniform GO/CNPs/GO sandwich structure. CNPs in membranes not only increase the distance between GO layers, but also form narrow 1D zigzag channels between 2D nanochannels to size-exclude dye molecules.

## 2 Experiments

### 2.1 Materials

All of the chemicals were used as they were obtained. HNO_3_ (Shanghai Lingfeng Chemical Reagent Co., Ltd, 65%), H_2_O_2_ (Sinopharm Chemical Reagent Co., Ltd, ≥30%), NaNO_3_ (Sinopharm Chemical Reagent Co., Ltd, ≥99%), H_2_SO_4_ (Sinopharm Chemical Reagent Co., Ltd, 98%), flake graphite (analytically pure, Qingdao Tianshengda graphite). Methylene blue (MB, Mw = 320), rhodamine B (RhB, Mw = 479), methyl orange (MO, Mw = 327), bromocresol green (BCG, Mw = 698), Evans blue (EB, Mw = 961), ethanol (Sinopharm Chemical Reagent Co., Ltd, ≥ 99.7%), NaCl (Macklin Co., 99.5%). All reagents were of analytical grade unless otherwise stated. PES membrane (pore size 0.1 μm) was purchased from Yibo Filter Equipment Factory in Haining City, Zhejiang; carbon ink (the concentration of carbon nanoparticles is 95.3 mg mL^−1^) was purchased from Shanghai Hero Co., Ltd. Deionised water was used throughout the experiment.

### 2.2 Preparation of membrane

Graphite oxide (GO) is produced from flake graphite by a modified Hummers' method^35,36^ and further dispersed in distilled water to obtain a GO solution with a concentration of 3 mg mL^−1^. For membrane construction, 0.5 mL of GO solution and a certain volume of carbon ink (0 μL, 0.75 μL, 1.5 μL, 3 μL and 6 μL) were added to 40 mL of deionised water and the mixture was sonicated for 30 min to form a homogeneous solution. The GO-based membrane was then fabricated by vacuum filtration on a mixed microporous PES support membrane (pore size 0.1 μm). The mass ratios of CNPs to GO in the composite membranes are approximately 0, 0.05, 0.1, 0.2 and 0.4, respectively. The membranes were then dried in an oven at 50 °C. The resulting membranes were named GO, GO/CNPs-5, GO/CNPs-10, GO/CNPs-20 and GO/CNPs-40, respectively.

### 2.3 Characterizations of membranes

The surface and cross-sectional morphology of the nanofiltration membrane was characterized using a scanning electron microscope (SEM, Hitachi S-4800). A suitably sized sample of the membrane was adhered to the conductive adhesive. It was then placed on the sample stage of the scanning electron microscope. To observe the cross section, the sample must be placed in liquid nitrogen to break it. All samples were gold-plated before testing and then scanned at 10 kV. Transmission electron microscopy (TEM, Talos F200X G2) was used to characterize GO and GO/CNPs composites, respectively. The sample to be tested was dispersed in anhydrous ethanol and prepared at a certain concentration. The sample was then prepared on the microgrid by the lifting and drying test method. The Raman spectra were measured on a Lab RAM Aramis Raman spectrometer (HORIBA Scientific Lab RAM HR Evolution, 50–4000 cm^−1^) with an excitation wavelength of 532 nm. The UV-visible adsorption spectra were recorded on a Shimadzu UV-2550 spectrophotometer. X-ray powder diffraction (XRD) was recorded using the scanning mode of the XRD-7000. The average interlayer spacing of the sample (NF membranes) was measured according to Bragg's law. Infrared spectroscopy was measured using an infrared spectrometer (PerkinElmer Spectrum two).

### 2.4 Evaluation of the membrane separation performance

Five typical dyes with different molecular weights, methylene blue (MB), rhodamine B (RhB), methyl orange (MO), bromocresol green (BCG) and Evans blue (EB), were selected at 10 mg L^−1^ to test the separation performance of the membrane on the dye. Evaluation of the performance of the manufactured nanofiltration membranes was performed by a vacuum filtration system (Fig. S1†). The suction filter device is composed of SHZ-D (III) circulating water multi-purpose vacuum pump, Feida sand core suction filter device (300 mL filter bowl, sand core filter head, 500 mL receiving bottle, clip), and silicone connecting pipe to filter at 25 °C.

In the experiment, the support membrane was polyethersulfone (PES) with a pore size of 0.1 μm. In each experiment, the effective area of the membrane sample was 12.56 cm^2^ (*A*). The tests were all performed at low pressure (∼0.1 MPa). Prior to evaluation, the membrane was pre-compressed at a pressure of 0.1 MPa for 30 min. Three replications were done to measure water flux. The flux (*J*, L m^−2^ h^−1^) of membrane was estimated by eqn (1).^22^1
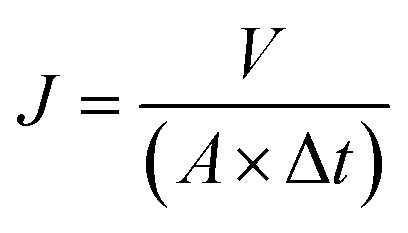
where *V* (L) is the volume of permeated water, *A* (m^2^) is the effective area of GO membrane, Δ*t* (h) is the water permeation time.

The concentrations of dyes in the feed and permeate were measured using UV-vis spectrophotometry. The rejection ratios can be calculated using the following eqn (2).^37^2
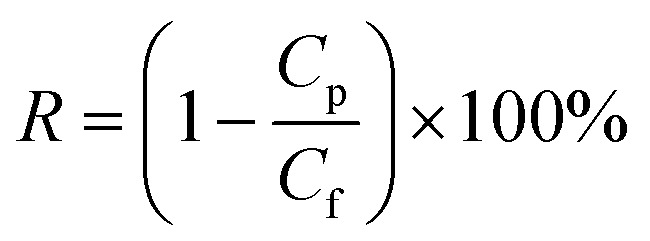
where *C*_p_ and *C*_f_ represent the solute concentration (mg L^−1^) of the permeates and feed solutions, respectively. To ensure the repeatability of the experimental results, each group of samples was subjected to at least three repeated separation performance tests.

In addition, a binary separation experiment was carried out on GO/CNPs composite membranes, and a mixture of 10 mg L^−1^ dye and 1000 mg L^−1^ NaCl was prepared in a mixed solution to test the rejection performance of the membranes under salt-dye coexistence conditions. Ion chromatography was used to test the Na^+^ concentration of the solution before and after filtration. The rejection ratios can be calculated using the following eqn (3).^38,39^3
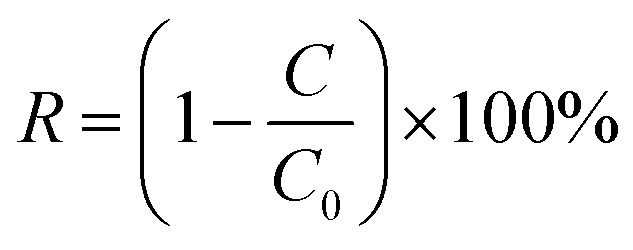
where *C* and *C*_0_ represent the solute concentration (mg L^−1^) of the permeates and feed solutions, respectively. To ensure the repeatability of the experimental results, each group of samples was subjected to at least three repeated separation performance tests.

### 2.5 Antifouling performance measurements

To measure the anti-fouling performance of the membrane, the test was carried out using bovine serum albumin (BSA) at a concentration of 500 ppm.^40^ First, DI water flux *J*_0_ of the membrane was determined after a 75 min pure water permeation test at 25C and 0.1 MPa. Then, The BSA solution of 500 ppm was filtered through the membranes, and *J*_1_ was recorded after every 15 min. After that, the membrane surface was flushed with distilled water for 30 min. Finally, the steady water flux of cleaned membrane *J*_2_ was measured again at the same operation condition. The flux recovery ratio (FRR) and the other fouling resistance parameters including total fouling ratio (*R*_t_), irreversible fouling ratio (*R*_ir_) and reversible fouling ratio (*R*_r_) were determined to investigate fouling behavior of the resulting membranes in more detail using eqn (4)–(7).^41^4
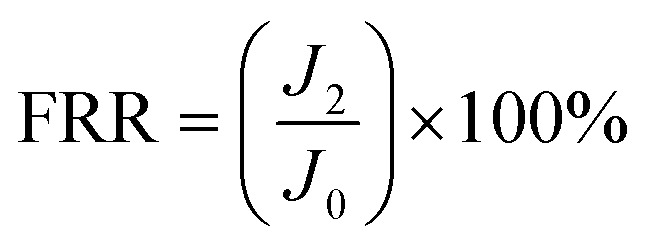
5
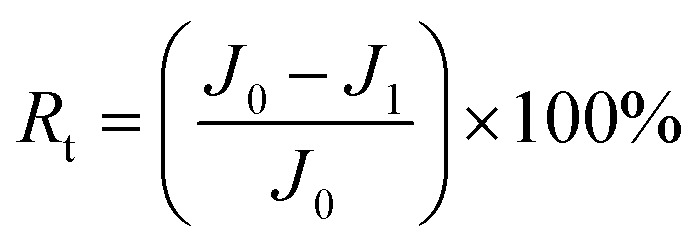
6
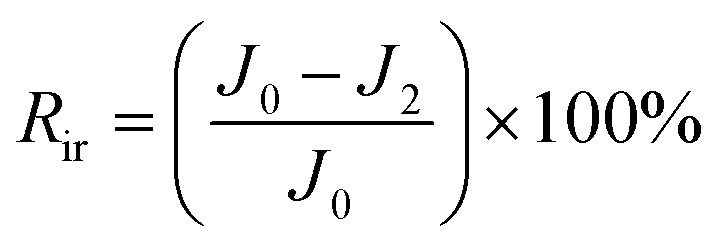

7 *R*_r_ = *R*_t_ − *R*_ir_

## 3 Results and discussion

### 3.1 Membrane construction

**Fig. 1:**
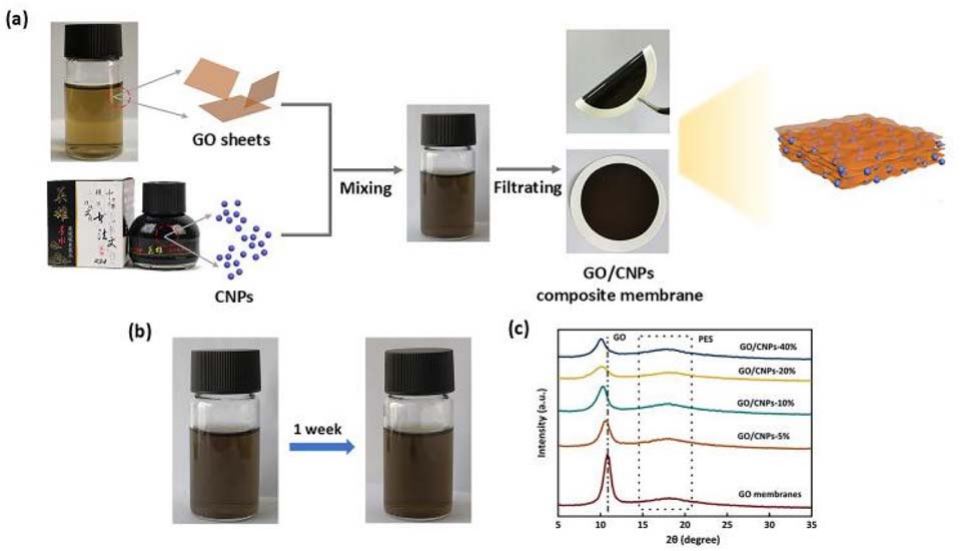
(a) Schematic diagram of GO/CNPs membrane synthesis process, (b) GO-CNPs mixed solution, (c) XRD of the prepared membranes.

The key to constructing a uniformly sandwiched GO membrane with CNPs is to obtain a stable and homogeneous precursor solution. It is well known that carbon ink is a highly stable nanocarbon dispersion, which is widely used in Chinese calligraphy and painting. In this work, carbon ink was used as a source of CNPs for the preparation of GO membranes with uniform sandwich structure. As shown in Fig. 1a, a simple strategy is designed to improve the GO membrane performance by sandwiching CNPs between GO layers. The mixture of GO and CNPs is first dispersed in aqueous media by ultrasonication to obtain a precursor solution. Note that a good GO-CNPs dispersion liquid can be kept in a stationary state for one week without any deposition (Fig. 1b). This is because they possess a zeta potential that is more negative than −30 mV (Fig. S2†).^42^ A GO/CNPs membrane is then prepared by filtration of the GO and CNPs dispersion mixture. In this process, the CNPs are inserted between the GO layers and play an important role in inhibiting the aggregation of 2D GO. Inserting the CNPs between the GO layers creates open channels for water transport and reduces the water diffusion length by preventing the GO sheets from aggregating, leading to a significant increase in water flow. A series of GO/CNPs-a membranes were obtained by adjusting the amount of CNPs added, where a represents the mass percentage of CNPs in the composite membrane.

X-ray diffraction (XRD) was used to analyze the changes in layer spacing of the GO resulting from the incorporation of different amounts of CNPs, as shown in Fig. 1c, the diffraction peak of pure GO (001) was located at 10.9° and the layer spacing was 0.81 nm. In the case of the GO/CNPs membranes, the characteristic peaks all showed a smaller angle shift, which was proportional to the amount of CNPs added. The characteristic XRD diffraction peaks associated with GO and CNPs are shown in Fig. S3.† According to the Bragg equation 2dsinθ = nλ,^43^ the values of the layer spacing *d* and the diffraction angle *θ* are inversely proportional, and the diffraction peak gradually shifts to a lower angle with increasing CNPs content, indicating that the layer spacing gradually increases. With a greater quantity of inserted carbon nanoparticles, the interlayer distance grows larger (Fig. S4†). For two-dimensional membranes, the space between adjacent nanosheets provides the main channel for molecular transport, making layer spacing an important parameter affecting the permeability of composite membranes.^44^ For GO/CNPs-20, the interlayer distance is increased to 0.87 nm, which is conducive to improving the water flow of the membrane.

### 3.2 Membrane morphology and structure

**Fig. 2:**
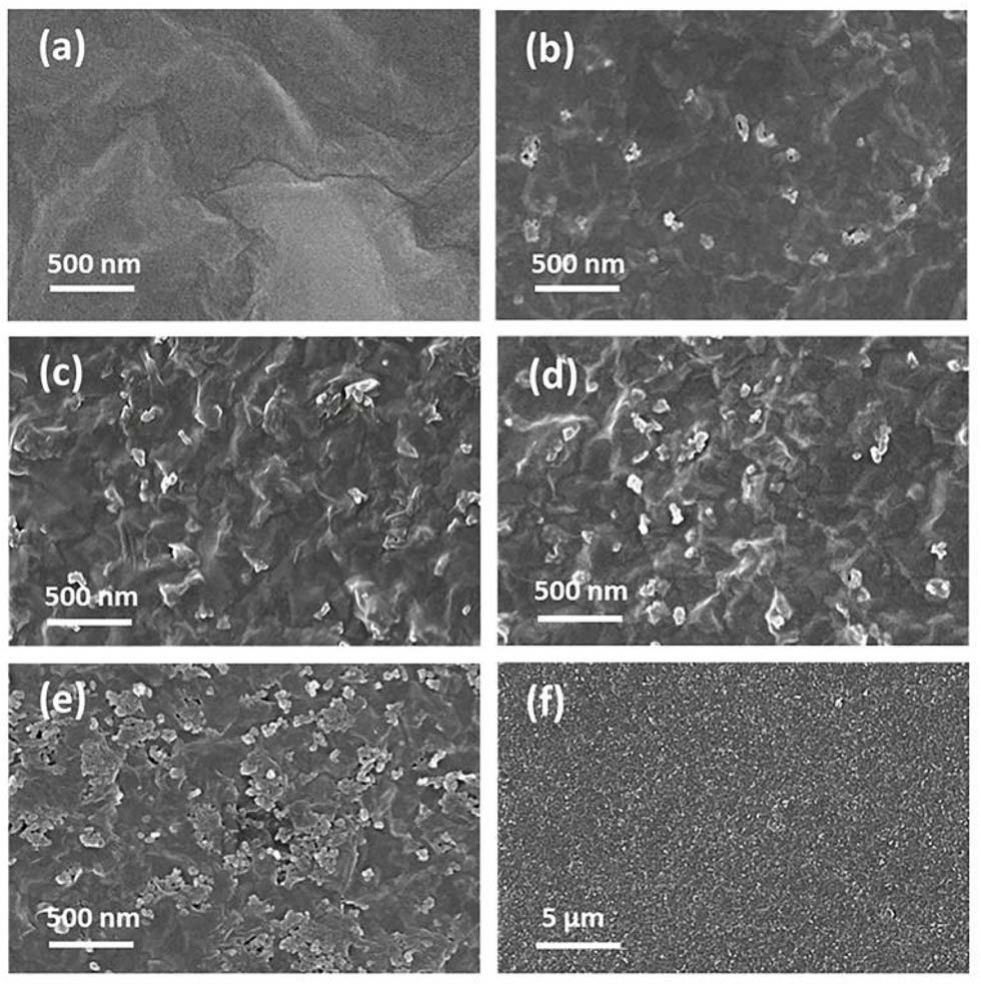
Surface morphology of membranes with various amounts of CNPs. (a) GO, (b) GO/CNPs-5, (c) GO/CNPs-10, (d) GO/CNPs-20, (e) GO/CNPs-40, (f) SEM image of a carbon nanoparticles.

The surface of the GO/CNPs composite membrane was characterized by SEM and the results are shown in Fig. 2. For the pure GO membrane (Fig. 2a), straight wrinkles can be observed, which is in good agreement with the TEM data (Fig. S4†). When CNPs were added, nanodimensional protrusions similar in size to pure CNPs (Fig. 2f and S5†) were formed on the membrane surface (Fig. 2b–e) which is attributed to CNPs covered by GO nanosheets (Fig. S5†). When the mass ratio of CNPs was increased to 0.4, some aggregates of CNPs and broken gaps could be seen on the membrane surface (Fig. 2e), indicating that a looser stacking structure was formed. Although the water permeability of GO/CNPs-40 composite membranes can be improved, the dye rejection of the membranes will be reduced. EDX elemental mapping analysis demonstrated that the main components of GO/CNPs-20 were carbon (C) and oxygen (O). These elements displayed an almost homogeneous distribution across the entire membrane surface (Fig. S6†), indicating the formation of uniform membrane structure. The high carbon concentration reflected the structural characteristics of graphene oxide and carbon nanoparticles. Meanwhile, the presence of oxygen indicated an ample amount of oxygen-containing functional groups on the surfaces of GO and CNPs.

**Fig. 3:**
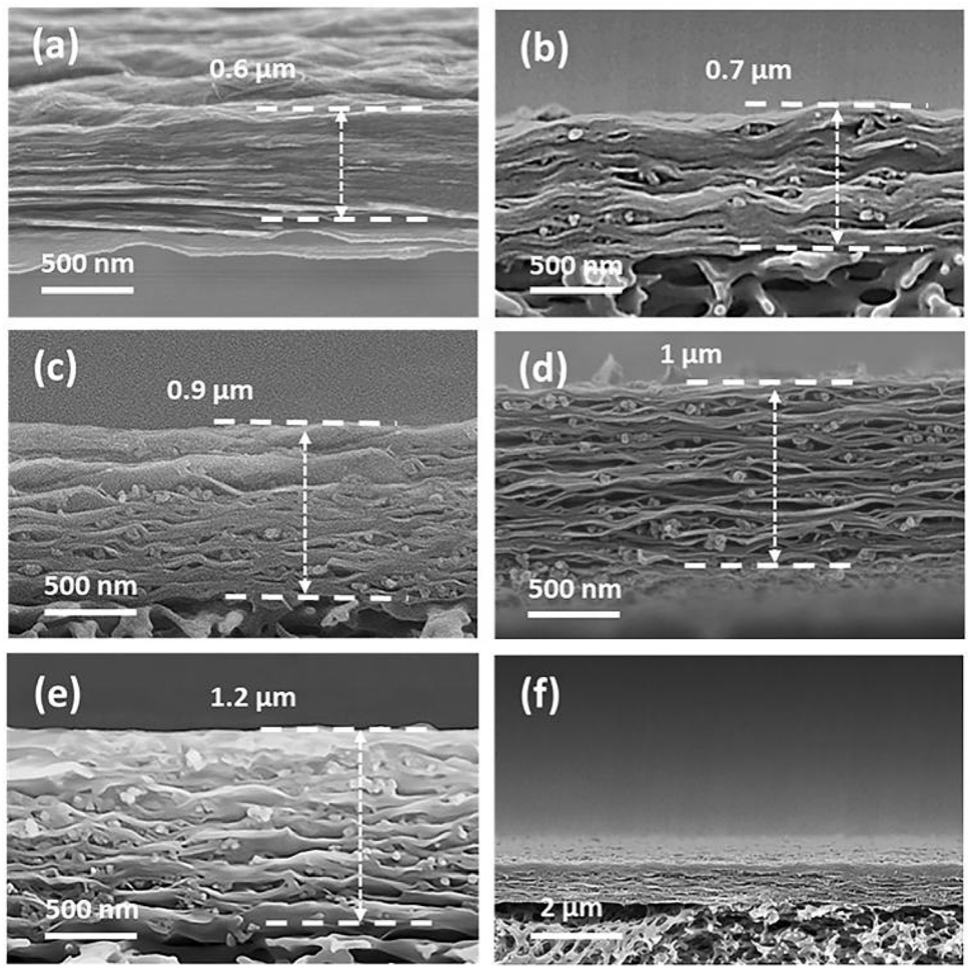
Cross-sectional SEM images of the membranes. (a) Pure GO, (b) GO/CNPs-5, (c) GO/CNPs-10, (d) GO/CNPs-20, (e) GO/CNPs-40, (f) overall morphology of the GO/CNPs-20 membrane.

To directly observe the CNPs sandwiched between the GO sheets, SEM images of the cross-section of the membranes were taken and shown in Fig. 3. The pure GO membrane showed a densely packed structure with a thickness of 0.6 μm (Fig. 3a). For the GO/CNPs composite membranes, the originally densely packed GO nanosheets were gradually embedded with carbon nanoparticles (Fig. 3b–e). It is worth noting that the distribution of nanoparticles in the GO/CNPs-20 membranes is impressively uniform (Fig. 3d), indicating that the CNPs and GO sheets are homogeneous throughout the filtration process. When the mass ratio of CNPs in the composite membrane was changed from 0 to 0.4, the composite thickness increased from 0.6 μm to 1.2 μm, showing that a looser layer structure is formed as more CNPs are inserted, which is consistent with the XRD result. In GO-based membranes, the space between adjacent nanosheets serves as the main channel for molecular transport.

**Fig. 4:**
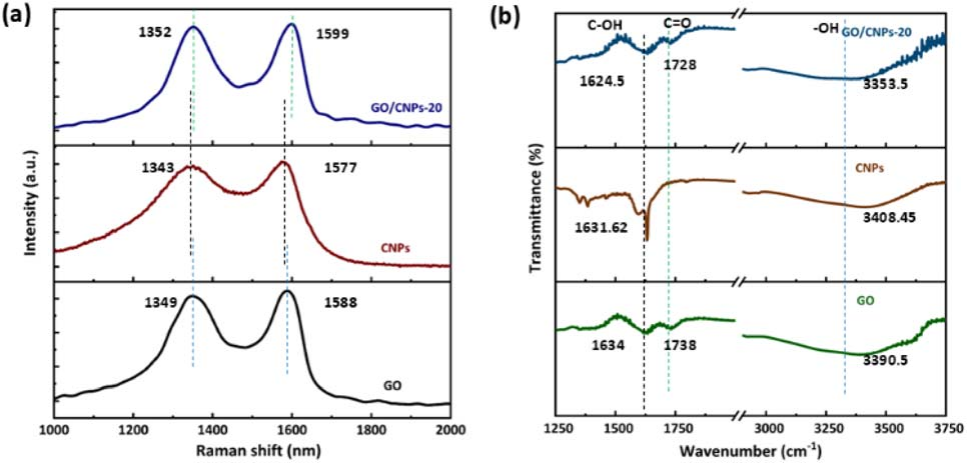
(a) Raman spectra of GO membranes, CNPs, GO/CNPs composite membranes, (b) infrared spectrogram of GO membranes, CNPs, GO/CNPs composite membranes.

Fig. 4 shows the Raman spectroscopy and Fourier transform infrared spectroscopy of pure GO membranes and GO/CNPs composite membranes. Obvious red shifts were observed in the GO/CNPs composite membranes compared to the pure GO membrane. For example, the D Raman band shifted from 1349 cm^−1^ to 1352 cm^−1^, while the G Raman band shifted from 1588 cm^−1^ to 1599 cm^−1^ (Fig. 4a), confirming the direct interaction between GO and CNPs (hydrogen bond).^45–48^ Furthermore, when the CNPs were introduced, the infrared spectra of the C–OH, C=O and –OH functional groups all shifted to lower wavenumbers (Fig. 4b). This is further evidence for the existence of a direct interaction between GO and CNPs.

### 3.3 Dye separation performance of the membrane

**Fig. 5:**
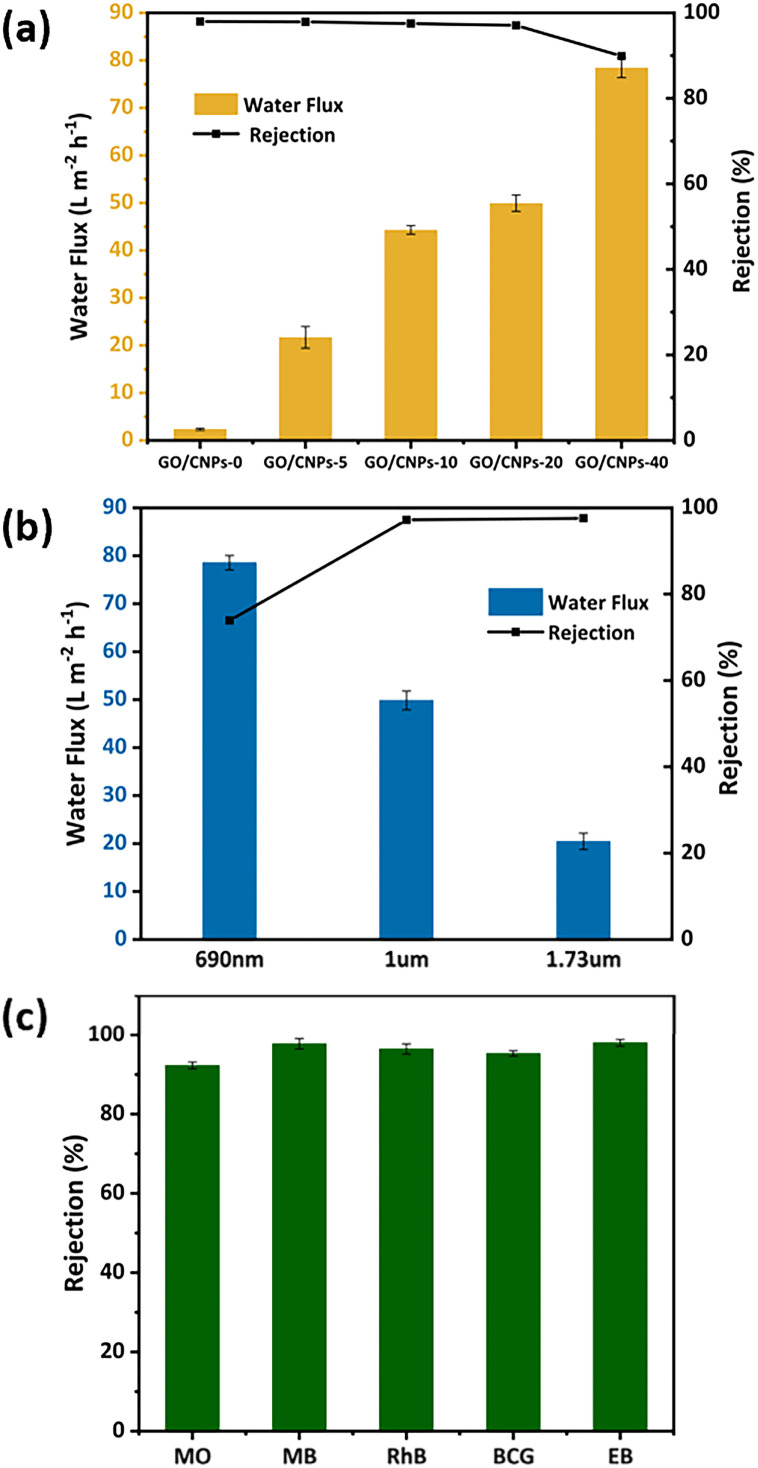
(a) Water flux and RhB (10 ppm) removal of membranes with CNPs/GO mass ratios of 0, 0.05, 0.1, 0.2, and 0.4, respectively; (b) water flux and RhB (10 ppm) removal of GO/CNPs-20 membranes with different thicknesses; (c) rejection of different dyes (10 ppm) by the GO/CNPs-20 membrane.

To investigate the effect of adding CNPs on separation performance, water fluxes and RhB rejections of composite membranes containing CNPs ranging from 5% to 40% were recorded and shown in Fig. 5a. Compared to the pure GO membrane, all composite membranes showed an increased water flow rate. For CNPs/GO-20, the maximum water flow rate was 49.9 L m^−2^ h^−1^, which was 20 times higher than the one of the pure GO membranes (2.3 L m^−2^ h^−1^). When the mass ratio of CNPs was further increased to 40%, the water flow of the composite membrane increased to 78.5 L m^−2^ h^−1^, which is comparable to that of GO/SiO_2_ membrane,^22^ and much higher than that of GQD-Ag/rGO membrane,^49^ and GO/CNTs membrane,^50^ (Table S1†). The above results indicate that there is a positive correlation between membrane flow rates and CNPs content, because more CNPs create more water channels, resulting in higher water permeability.

In addition to the flow rate of water, the rejection of target molecules is another important parameter for nanofiltration membranes. The RhB rejection of GO, GO/CNPs-5, GO/CNPs-10, GO/CNPs-20 and GO/CNPs-40 membranes is 98%, 97.9%, 97.5%, 97.1% and 89.9%, respectively. It can be concluded that the composite membranes with a CNPs content of less than 20% showed a rejection rate similar to the one of pure GO membrane. However, when the CNPs content was increased to 40%, the rejection rate significantly reduced to 89.9%. This result may be due to the local agglomeration of CNPs nanoparticles leading to the fracture of the GO layers as shown in Fig. 2e. In conclusion, the sandwiched CNPs nanoparticles could increase the water permeability of the membrane, but this had little effect on the target molecule rejection rate. Nevertheless, excessive CNPs may disrupt the intrinsic superposition structure of the GO nanosheet assembly and reduce the selective separation performance of the composite membranes. Since GO/CNPs-20 exhibited both an obviously increased water flow and high rejection rate, the mass ratio of CNPs to GO was selected to be 0.2, and GO/CNPs-20 composite membranes with different thicknesses were prepared to investigate the effect of film thickness on performances (Fig. S7†). The thickness of the GO/CNPs-20 membrane can be easily controlled from 0.69 μm to 1.73 μm by varying the volume of mixed dispersion during vacuum filtration. For the membrane prepared with 0.25 mL of CNPs and GO dispersion, the water flow is higher than the one prepared with 0.5 mL of CNPs and GO dispersion although the rejection rate is lower than the one prepared with 0.5 mL of CNPs and GO dispersion. The water flow rate decreased from 78.9 L m^−2^ h^−1^ to 20.4 L m^−2^ h^−1^ when the volume of CNPs and GO dispersion was increased to 1 mL. Based on the above results, the ideal ratio of CNPs to GO was found to be 0.2 and the ideal volume of CNPs and GO dispersion was found to be 0.5 mL.

Molecular selectivity is one of crucial parameters for the nanofiltration. Here we select five kinds of dye molecules with different weights (319.85–960 Da) including methylene blue (MB), rhodamine B (RhB), methyl orange (MO), bromocresol green (BCG), and Evans blue (EB) comparison. Their chemical structures, surface charge, and molecular sizes are given in Table S2.† As shown in Fig. 5c. The GO/CNPs-20 membrane still exhibited remarkable high rejections of >95% for MB, RhB, BCG, and EB at high water permeances of 47 L m^−2^ h^−1^. However, in the case of MO with small molecular size of 1.13 × 0.42 nm, the GO/CNPs-20 membrane show 92% rejection as a result of low effective sieving effects for smaller molecules. Furthermore, when the dye concentrations varied from 10 to 50 ppm, the rejection rate of GO/CNPs-20 membrane for MB was 96.5–97.8% (Fig. S8†), which was higher than that of RhB. This is because there were two main interactions between MB and GO, namely electrostatic interaction (EI) and π–π conjugation.^51,52^ When the dye concentration was not very large, the adsorption effect of the membrane also contributed to the retention of MB.^51^ We measured the zeta potential of the membrane and found that the membrane exhibited a significantly negative charge characteristic within the pH range of 3–11 (Fig. S9†). Meanwhile, we tested the dye rejection experiments of the membrane under different pH conditions and found that the rejection rate under acidic conditions was higher than that under alkaline conditions, and the rejection rate of cationic dyes was higher than that of anionic dyes (Fig. S10†).

### 3.4 Antifouling characteristics of the prepared membranes

Membrane fouling refers to the treatment process where microorganisms, inorganic colloids and proteins are the main contaminants. Contaminants gradually accumulate on the surface of the filter membrane, resulting in a decreased permeate flow and an increased pressure requirements and maintenance costs.^53^ Studies have shown that hydrophilicity and membrane surface roughness are the two main factors affecting the antifouling performance of membranes.^54,55^ A more hydrophilic and smooth membrane surface is expected to prevent the settling and building-up of dirt, resulting in better antifouling properties. In this work, the anti-fouling performance of the membranes has been investigated by using a BSA solution as a model protein fouling agent. The change in water flow over time in GO and GO/CNPs-20 membranes is shown in Fig. S11a.† The test was performed in three phases: the pure water flow test lasted 0–75 min, and the water flow test time converted to BSA (500 ppm) solution was 75–210 min. Finally, after rinsing the membrane, the pure water flow test was performed again with a test time of 210–315 min. The operating pressure conditions throughout the experiment were approximately 0.1 MPa. Data was collected every 15 min. When the feed was switched from dewatering to BSA solution, the permeability of the GO membrane and the GO/CNPs-20 composite membrane decreased rapidly. The water flow rate then reached a stable value after 15 minutes and continued to fluctuate slightly. The water flow of the cleaning membrane was restored to some extent after being washed but, the water flow rate of the washed membrane did not return to the rate of the original membrane. This is due to membrane fouling caused by the membranes filtered through the BSA solution. Typically, the high value of FRR and *R*_r_ and the low value of fouling indexes of *R*_t_ and *R*_ir_ are the proofs of the excellent anti-fouling performance of the NF-membrane.^41^ To analyze the fouling process in detail, we calculated the FRR, *R*_t_, *R*_r_, *R*_ir_ of the membranes and their values are shown in Fig. S12b.† The FRR values of GO and GO/CNPs-20 films were 71% and 72%, respectively, indicate that both have good antifouling properties. The *R*_t_ values were 50% and 48.5%, respectively. The *R*_r_ and *R*_ir_ of GO film were 21.4% and 28.6%, respectively, and the *R*_r_ and *R*_ir_ of the GO/CNPs-20 film were 20.9% and 27.6%, respectively (Fig. S12b†). In conclusion, the intercalation of CNPs nanoparticles had no significant effect on the antifouling performance of the membrane. The fouling resistance of membranes is affected by physicochemical and morphological properties, including the roughness, hydrophilicity, and charge of the membrane surface.^56^ A more hydrophilic and smooth membrane surface is expected to stop the deposition and accumulation of dirt, resulting in better antifouling properties. Contact angle measurements indicated that the introduction of carbon nanoparticles decreased the membrane contact angle from 41° to 39°. Thus, the intercalation of CNPs improves the hydrophilicity of the membrane, which is conducive to the improvement of the antifouling performance of the membrane. However, with the introduction of CNPs, the surface roughness of the film increased slightly (Fig. 2). Impurities tend to accumulate in the valley between CNPs. In summary, the antifouling performance of the membrane has hardly changed after the CNPs are embedded.

### 3.5 Membrane stability

**Fig. 6:**
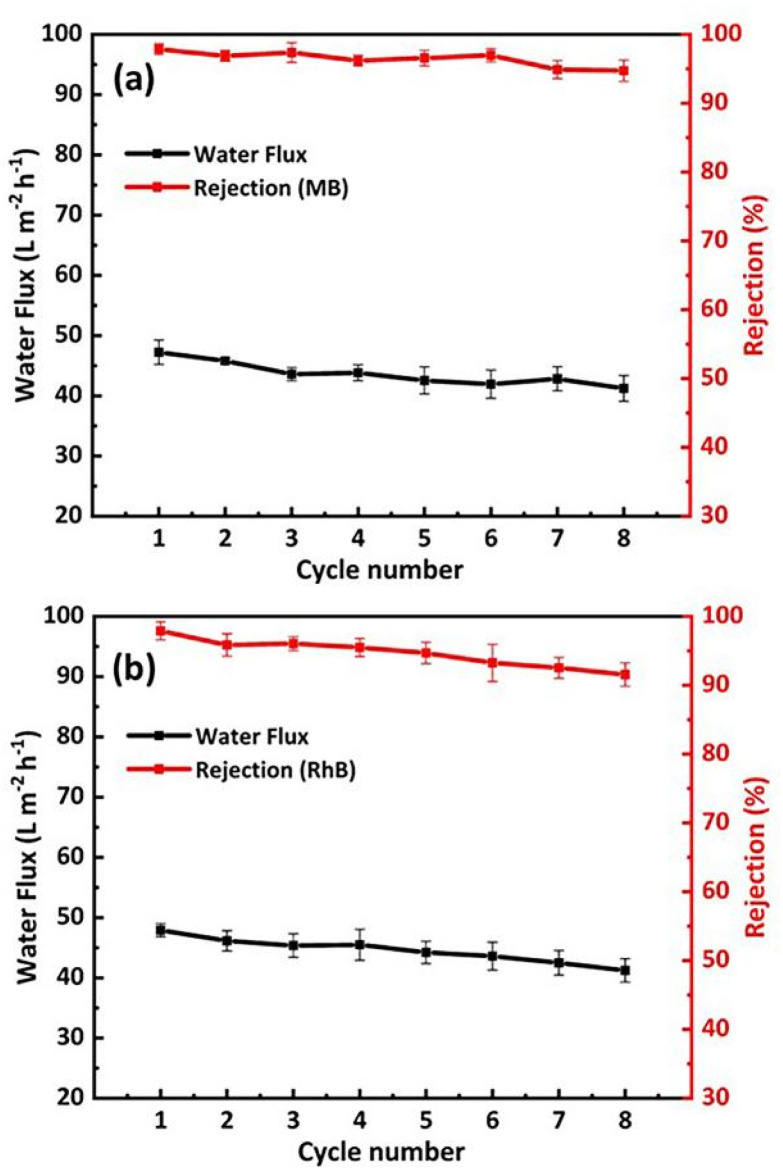
Long-term stability determination of GO/CNPs-20 membranes. (a) Methylene blue (MB) solution, (b) rhodamine B (RhB) solution.

In practical applications, the long-term stability of nanofiltration membranes is very important. Two dye solutions, MB (10 ppm) and RhB (10 ppm), with an operating pressure of approximately 0.1 MPa, were used to test the long-term stability of the GO/CNPs-20 membrane, as shown in Fig. 6. During the test, the sampled GO/CNPs composite membrane was washed with ethanol to remove residual dye from the membrane before further testing. After 8 consecutive cycles, the retention rate of MB gradually decreased from 97.8% to 94.7% and the retention rate of RhB gradually decreased from 97.9% to 91.6%. GO and CNPs form composite films through hydrogen bonding, physical adsorption and mechanical blocking. After several repeated washes, the membrane structure may change to some extent, resulting in a change in rejection rate. In addition, the water flow of the composite membrane remained variable (47.3–41 L m^−2^ h^−1^). In summary, the above results show that the GO/CNPs-20 membranes have a degree of stability and sieving effect. It has a good filtration effect on organic matter of a given molecular weight or particle size (such as MB and RhB dye molecules). Since the actual resistance of the membrane material to extreme pH is crucial, we carried out the separation performance test of GO/CNPs-20 membrane against MB solution with pH ranging from 3 to 11. The membrane still showed high mechanical stability under different pH conditions, and the rejection rate for MB was over 97% (Fig. S13a†). The stability of GO/CNPs-20 membrane was further confirmed by immersing it in a variety of solvents, including deionized water, acids and alkaline solutions. The membrane is robust and highly stable in acidic, alkaline and deionized water (Fig. S13b† and c).

### 3.6 Separation performance of graphene oxide composite membrane for binary components

**Fig. 7:**
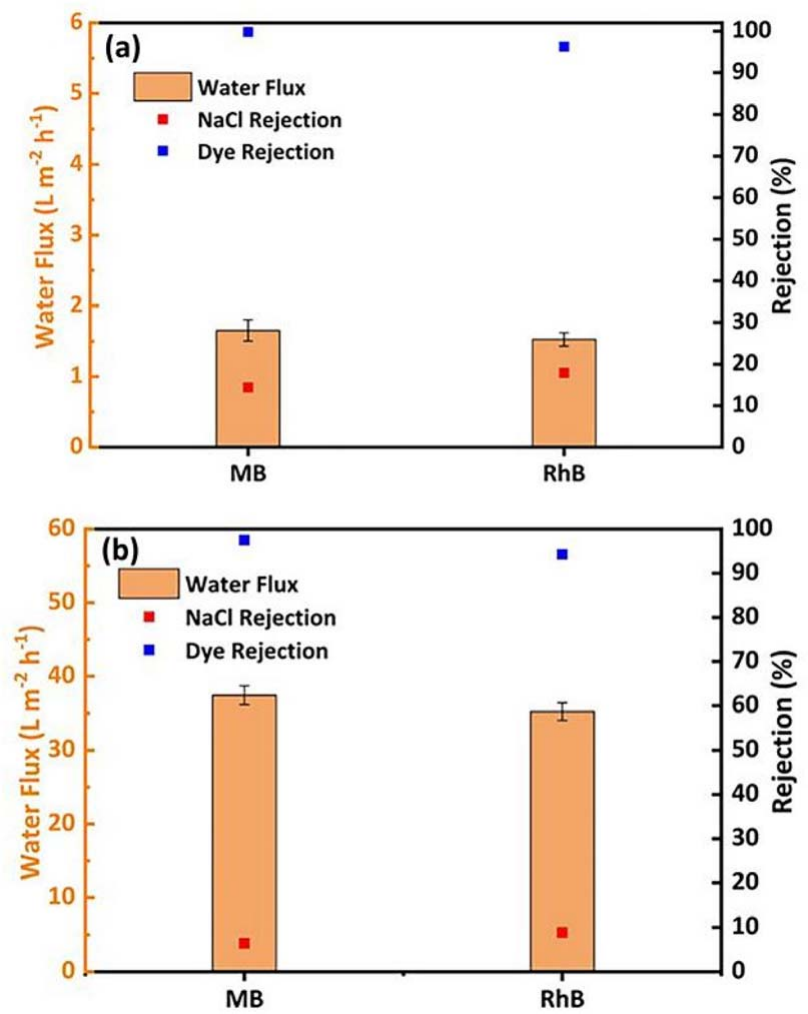
Separation of binary components. (a) The separation ability of GO membrane for MB/NaCl and RhB/NaCl mixed solutions, (b) separation capacity of GO/CNPs membranes for MB/NaCl and RhB/NaCl mixtures.

The separation of salts and dyes was investigated using a mixture of 10 ppm MB and RhB and 1000 mg L^−1^ NaCl, as shown in Fig. 7. The results in Fig. 7a show that the GO membrane has a rejection rate of 98.8% for dye, 14.4% for NaCl, 95.3% for dye and 17.9% for NaCl in RhB solution and a very low GO membrane flow of 1.6 L m^−2^ h^−1^ (MB solution) and 1.5 L m^−2^ h^−1^ (RhB solution) due to the compression of the lamellae at high salt concentrations. The GO membrane needs to be modified to improve the efficiency of the salt/dye separation process. According to the results in Fig. 7b, the rejection rate of GO/CNPs-20 in the MB mixed solution was 97.5% and the rejection rate of NaCl was 6.4%. The rejection rate of RhB was 94.3% and 8.8% in the RhB mixed solution. The addition of salts causes a slight decrease in the retention of dye molecules by the membrane. This may be due to a decrease in the repulsion of the Domnan effect.^57^ Furthermore, the filtration flow of the MB solution 37.4 L m^−2^ h^−1^ and the filtration flow of the RhB mixed solution was 35.2 L m^−2^ h^−1^, which were slightly lower than the flow rates of GO/CNPs-20 membranes in the pure dye filtration test, and the salt ion concentration was the most important factor affecting the filtration flow of the binary mixed solution. As the effect of NaCl weakens the agglomeration of dye molecules, uniformly dispersed dye molecules increase the risk of membrane penetration and form a denser cake layer on the membrane surface, resulting in a further decrease in flux.^58^ The experimental performance of the binary separation of MB- and RB-solution demonstrated the ability of the GO/CNPs-20 to effectively treat printing and dye effluents and to achieve salt recovery.

## 4 Conclusions

In conclusion, we have demonstrated the simple construction of GO-based nanofiltration membranes with uniform a sandwich structure by one-step vacuum-assisted filtration of carbon ink and GO mixed solution. Due to the stable and homogeneous precursor solution, CNPs were uniformly sandwiched between GO sheets, which appropriately increased the interlayer distance of the membranes. While having little effect on target molecule rejection, the sandwiched CNPs significantly increase water flow rate. The filtration test showed that the pure water flow of the GO/CNPs-20 composite membrane was 49.9 L m^−2^ h^−1^, which has been increased 21 times compared to a pure GO membrane. The rejection rate for four different organic dyes exceeds 97%, which is similar to the pure GO membrane. After 8 recycling cycles, the rejection rate for MB was still 94.7%. Moreover, the membranes allow the penetration of salts, which makes them promising for the desalination of dye effluents. Therefore, this work shows that introducing CNPs between the layers of GO nanosheets is an effective means to improve the performance of graphene-based nanofiltration membranes.

## Data availability

All relevant data are within the manuscript and its ESI.†

## Author contributions

Xue Zhang performed the membrane preparation, separation test and writing – original draft. Ziyi Sang and Leiyang Xue conducted the membrane characterization. Lianwen Zhu initiated the research, supervised the project, and reviewed the whole manuscript.

## Conflicts of interest

There are no conflicts to declare.

## Supplementary Material

RA-015-D5RA00454C-s001
