# Supplementary material for: One-step construction of carbon nanoparticle/graphene oxide nanofiltration membranes with uniform sandwich structure for enhanced water purification
Source: RSC Adv. 2025 Mar 28;15(13):9618–26. doi: 10.1039/d5ra00454c (PMC11952112; doi:10.1039/d5ra00454c)
Supplement: RA-015-D5RA00454C-s001 [file RA-015-D5RA00454C-s001.pdf]

Supplementary Information (SI) for RSC Advances.
This journal is © The Royal Society of Chemistry 2025

Supplementary Information

One-step construction of carbon nanoparticle/graphene oxide nanofiltration membrane with uniform sandwich structure for enhanced water purification

Xue Zhang,[a,b] Ziyi Sang,[b] Leiyang Xue [b] and Lianwen Zhu*[b]

a.College of Chemistry and Materials Science, Zhejiang Normal University, Jinhua, 321004, Zhejiang, China.

b.School of Biology and Chemical Engineering, Jiaxing University, Jiaxing, 314001, Zhejiang , China.

E-mail: lwzhu@zjxu.edu.cn

Synthesis method of graphene oxide:

Graphene oxide was prepared in this experiment by the modified Hummers method. Weigh 1.00 g of flake graphite and 0.750 g of $NaNO_3$, and measure 34 mL of H_2SO_4. Add them successively to a 250 mL beaker and stir thoroughly. Then weigh 5.00 g of $KMnO_4$ and add it to the previous mixture in 5 to 10 portions, with an interval of about 1 minute each time. The mixture should be placed in an ice water bath. After the prepared sample is left to stand at room temperature, stir it for 2 hours at 40°C in an oil bath. Then add 50 mL of deionized water in several portions. The reaction releases heat. After the liquid cools down, add 4 mL of H_2O_2 solution at one time. At this point, the liquid turns golden yellow. Then dilute the above liquid to 500 mL and centrifuge it to neutrality. After ultrasonic treatment for 6 hours, centrifuge the obtained dispersion at 6000 r/min. Take the middle layer of the suspension and remove the black substance at the bottom to obtain a stable and uniform graphene oxide dispersion.

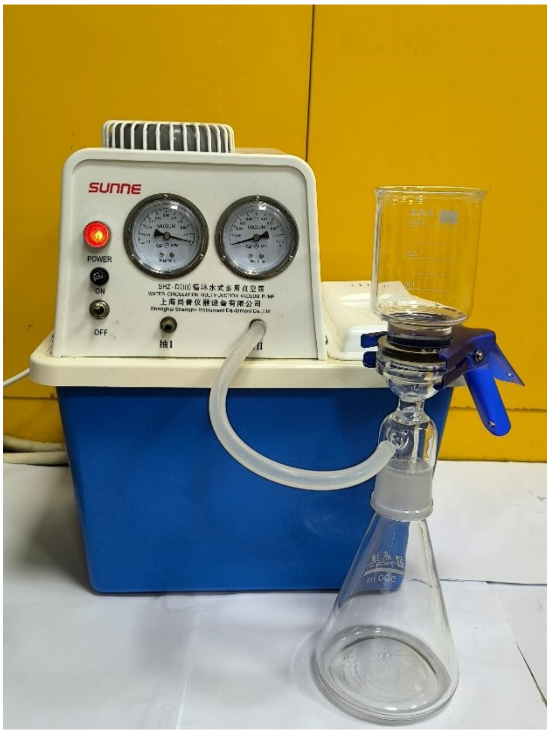

Fig. S1. Vacuum filtration system: The suction filter device is composed of SHZ-D(III) circulating water multi-purpose vacuum pump, Feida sand core suction filter device (300mL filter bowl, sand core filter head, 500mL receiving bottle, clip), and silicone connecting pipe.

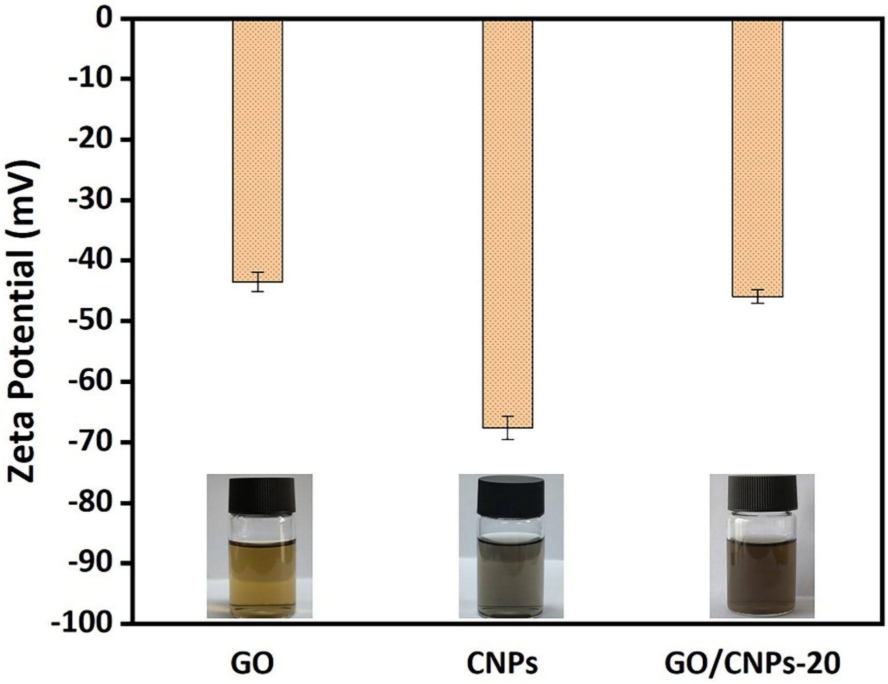

Fig. S2. Zeta potentials of GO, CNPs and GO/CNPs-20 solutions.

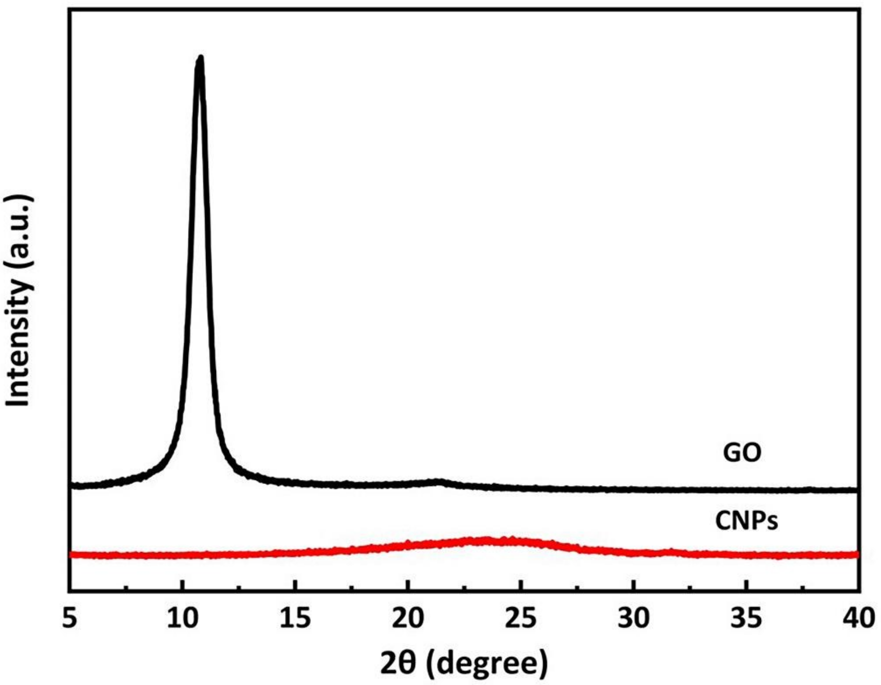

Fig. S3. XRD characteristic diffraction peaks of GO and CNPs.

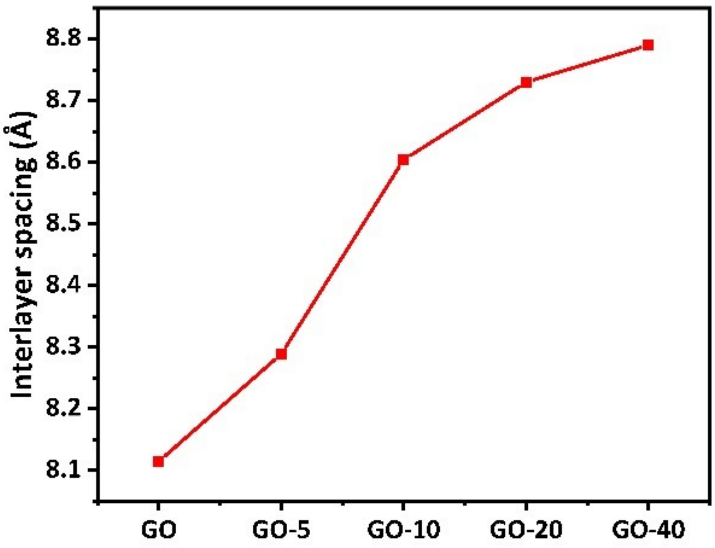

Fig. S4. The interlayer spaceing of membranes with various amounts of CNPs.

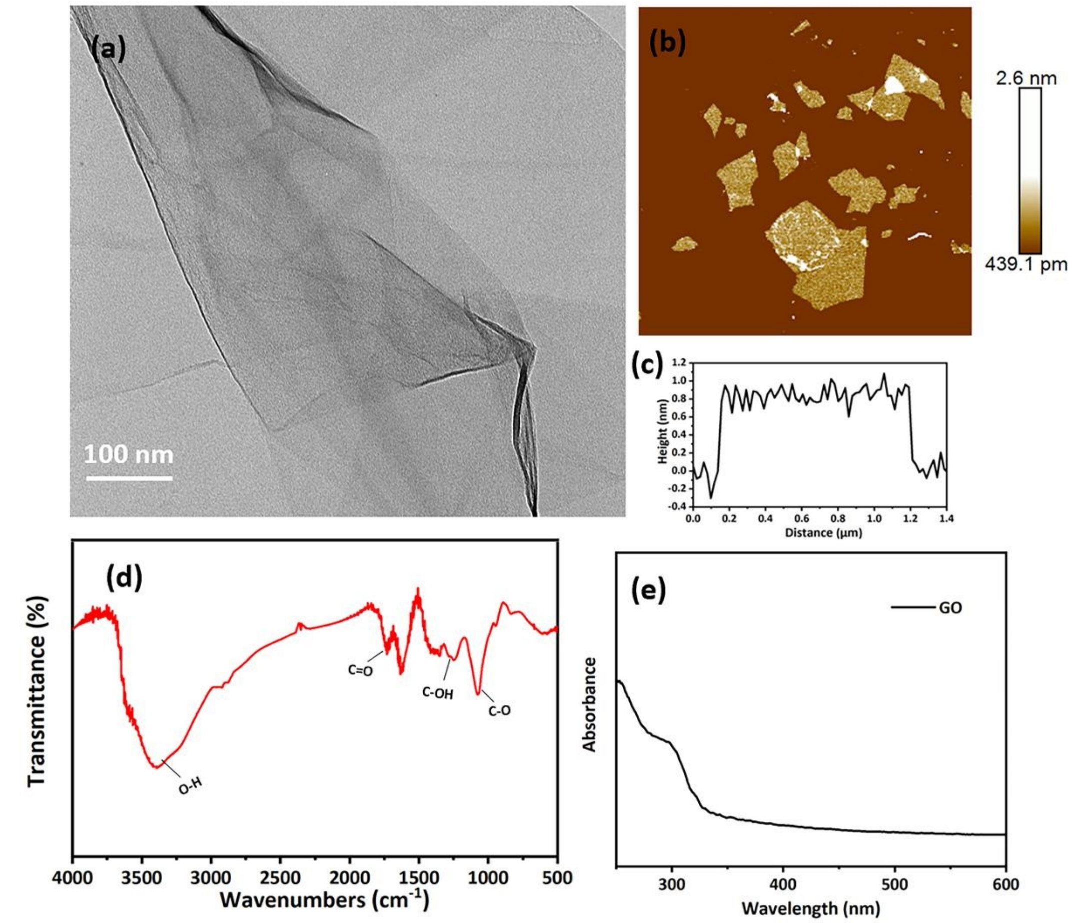

Fig. S5. Characterization of graphene oxide: (a) TEM image; (b, c) AFM image and corresponding height profile; (d) FTIR spectra; (e) UV-vis absorption spectra.

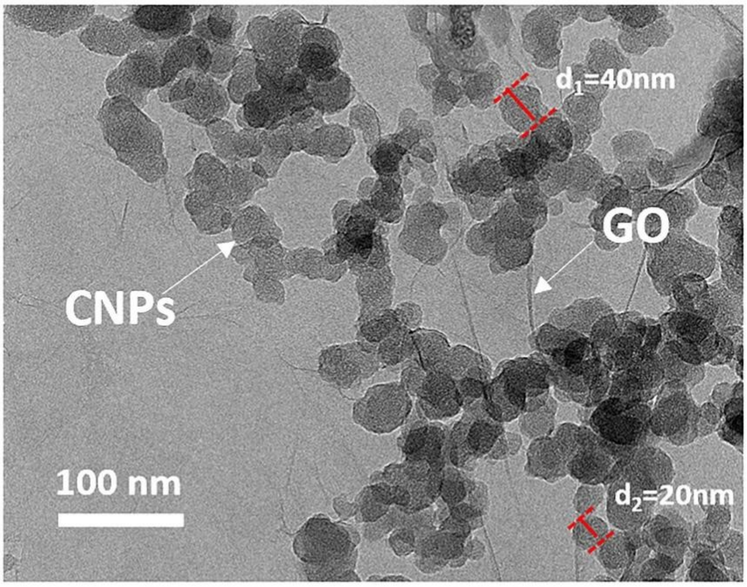

Fig. S6. TEM of the GO/CNPs composite membrane.

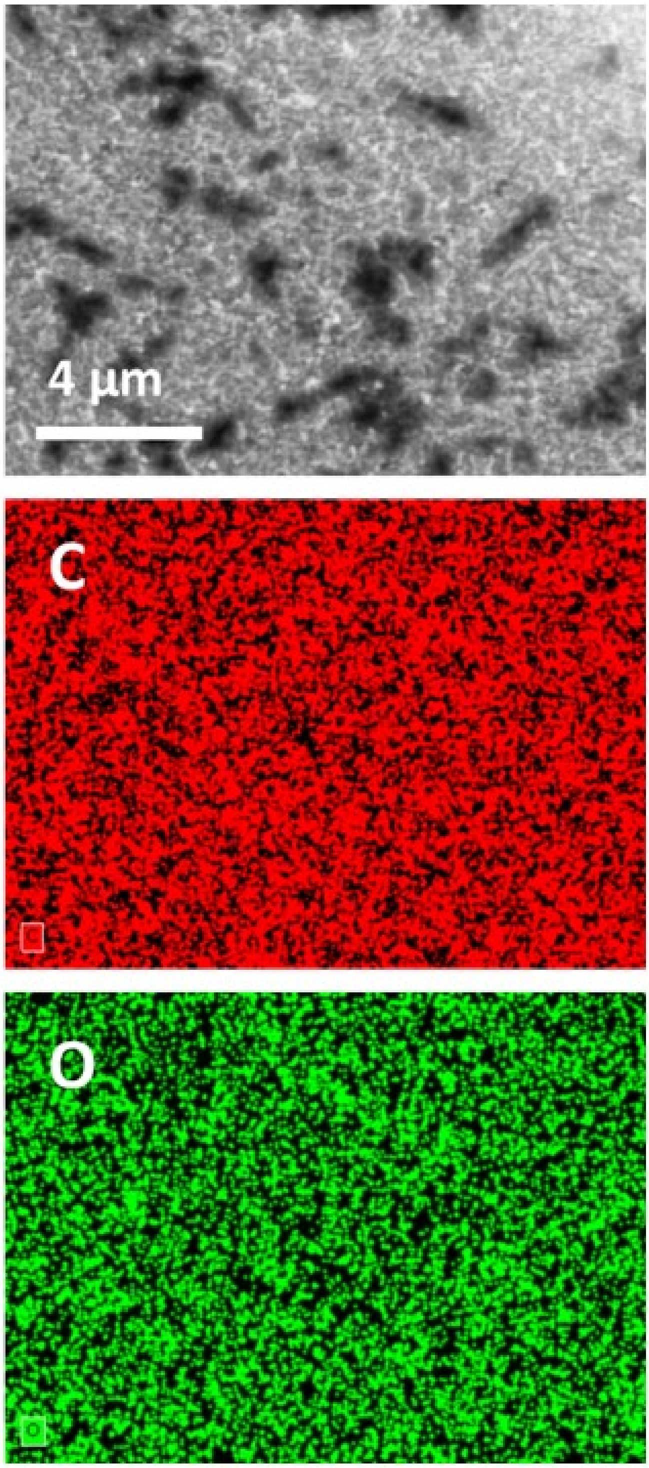

Fig. S7. The EDX mapping images of the GO/CNPs-20 composite membrane.

Table S1. Water flux of various GO-based separation membranes

Membrane	Water flux ($L \cdot m^{-2} \cdot h^{-1}$)	References
GO/CNPs (40 wt%)	78.5	This work
GO/SiO_2 (30 wt%)	72.8	Purif. Technol., 2021, 278, 119440.
rGO/Fe_2O_3	24.90	Desalination, 2024, 587, 117919.
Zr-Porphyrin@PG	29.2	Appl. Surf. Sci., 2025, 687, 162290.
GQD-Ag/rGO	1.36	Chem. Eng. J., 2023, 465, 143005.
GO/CNTs	34.4	J. Water Process. Eng., 2021, 40, 101901.
GO/TANs	52.1	J. Membr. Sci., 2023, 686, 122027.

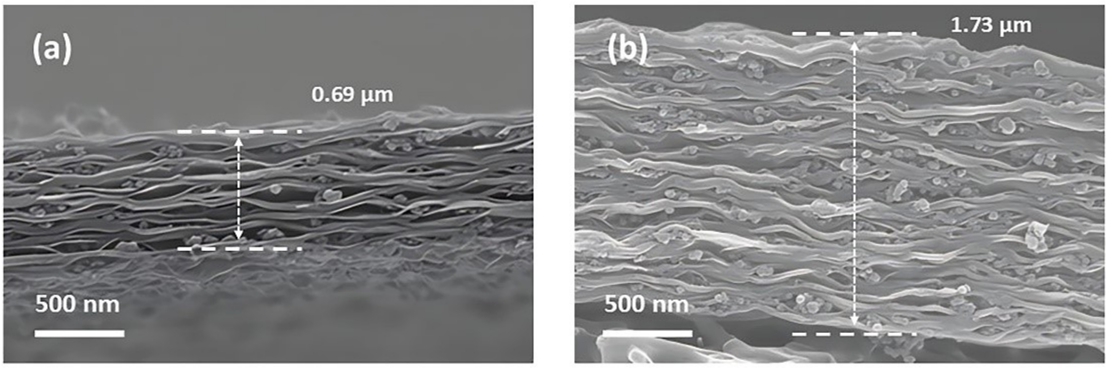

Fig. S8. Cross-sectional SEM images of GO/CNPs-20 composite membranes: (a) GO/CNPs-20 membranes prepared with 0.25 mL of CNPs/GO dispersions (b) GO/CNPs-20 membranes prepared with 1 mL of CNPs/GO dispersions.

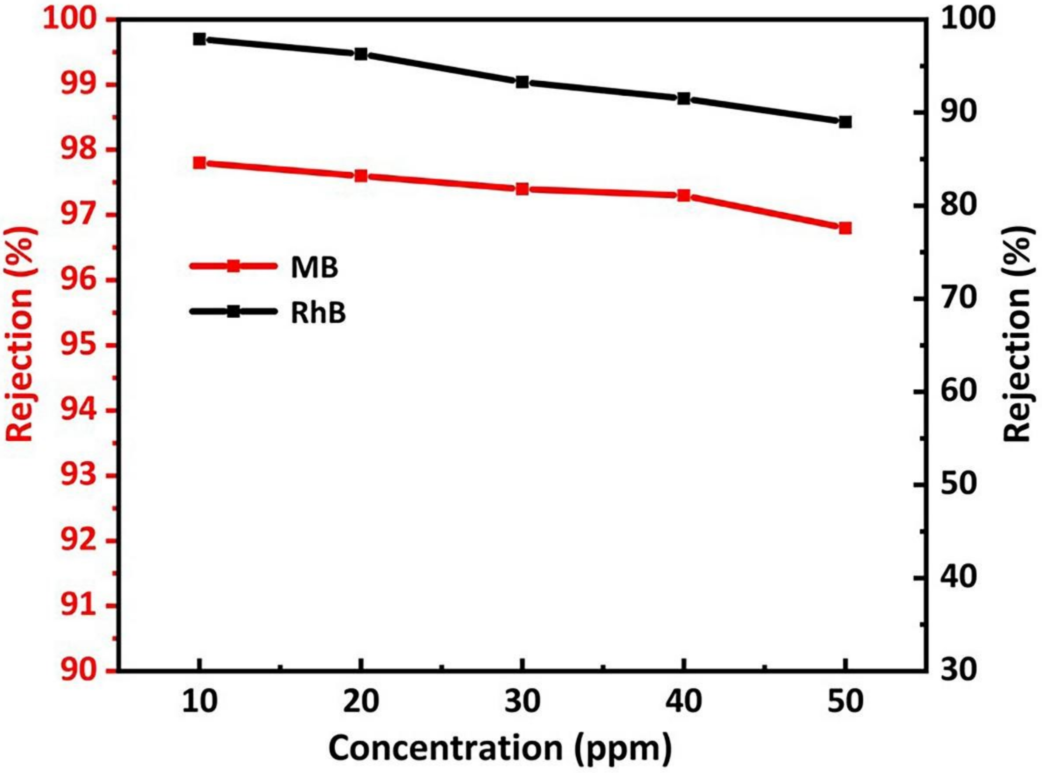

Fig. S9. Variation of dye (RhB and MB) rejection rate with different dye solution concentration for GO/CNPs-20 membrane.

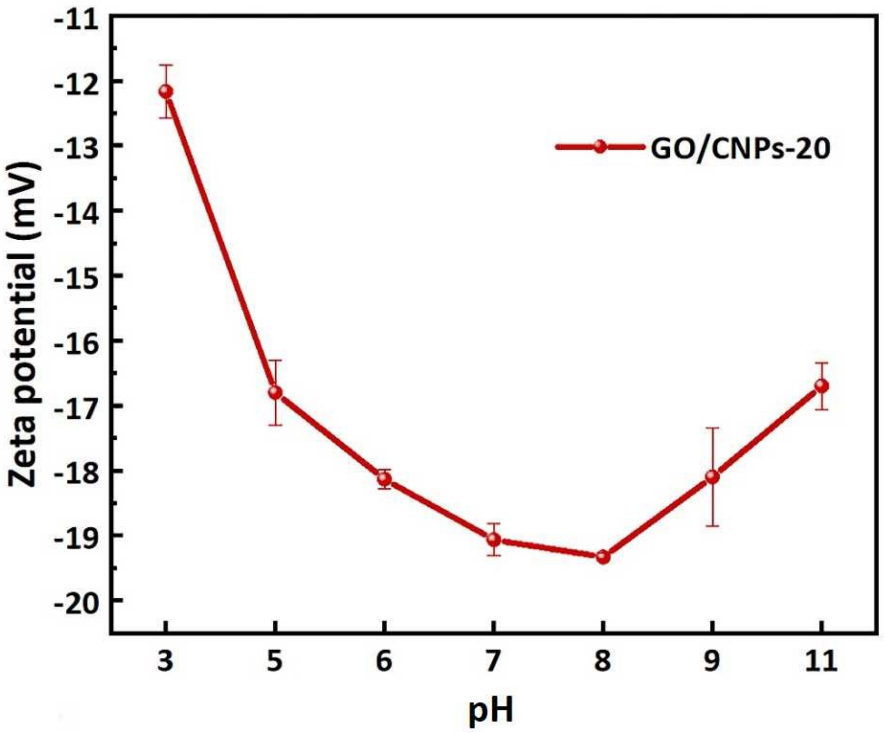

Fig. S10. Zeta potential of the GO/CNPs-20 membrane at various pH.

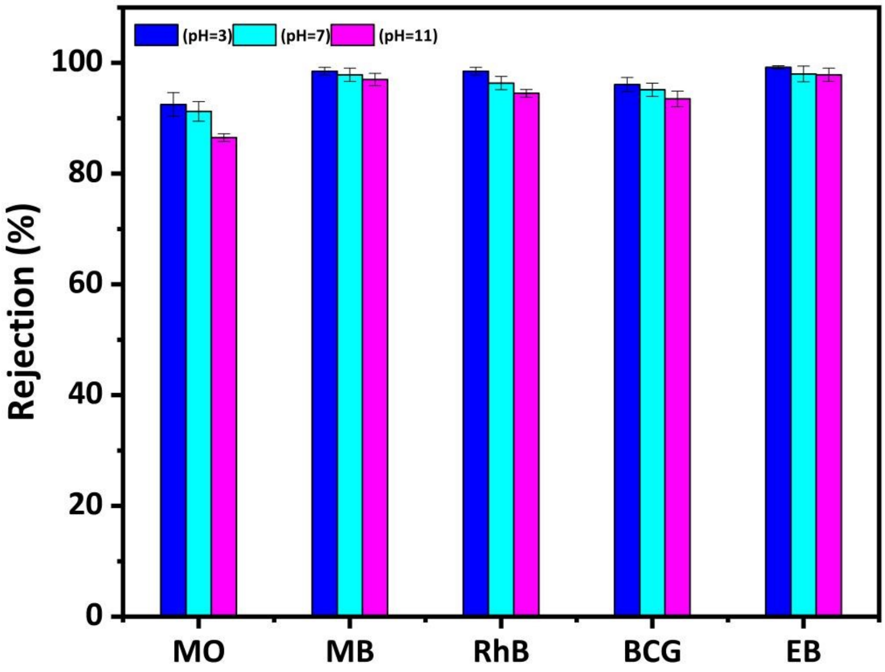

Fig. S11. Dye rejection of the GO/CNPs-20 membrane at various pH.

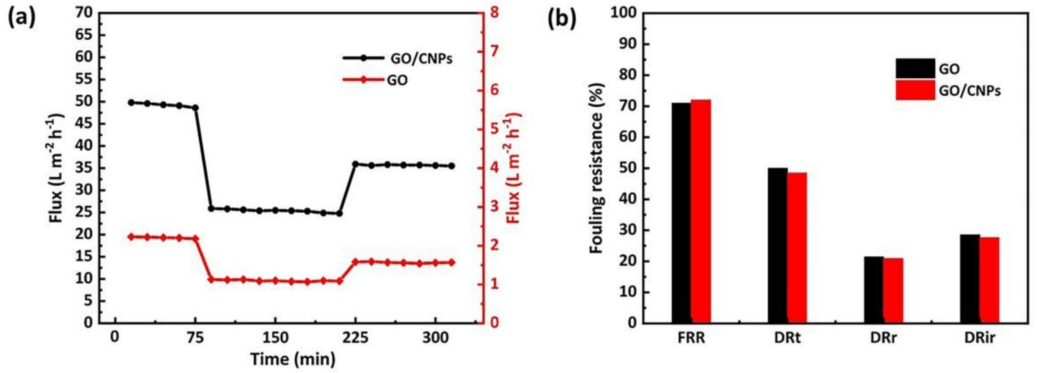

Fig. S12. Antifouling performance of the membranes. (a) Water flux as a function of testing time; (b) Fouling resistance ratio of the GO/CNPs-20. The tests were carried out for three periods: 0-75 min for pure water flux, 75-210 min for the water flux in BSA (500 ppm) solution and 210-315 min for pure water flux of the membranes after washing.

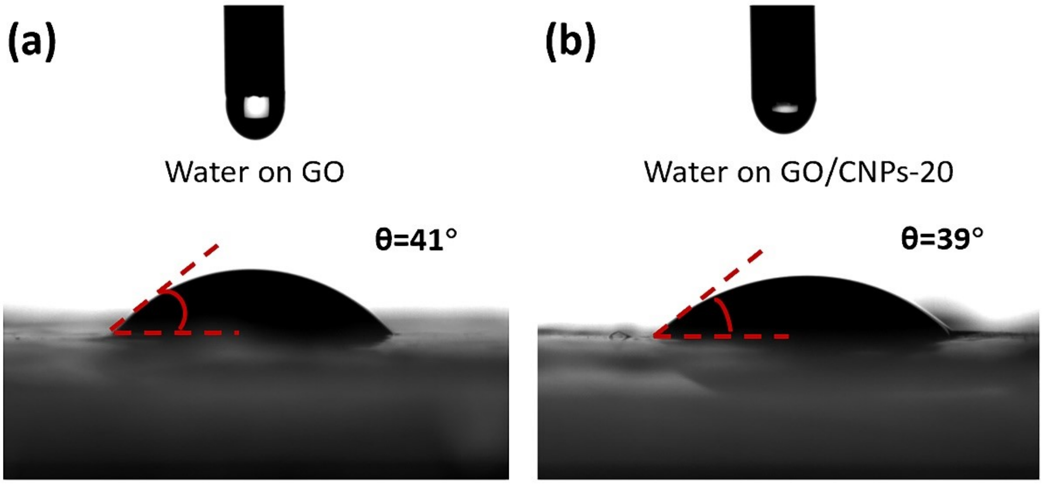

Fig. S13. Contact angle test of the membrane (a) GO membrane (b) GO/CNPs-20 membrane.

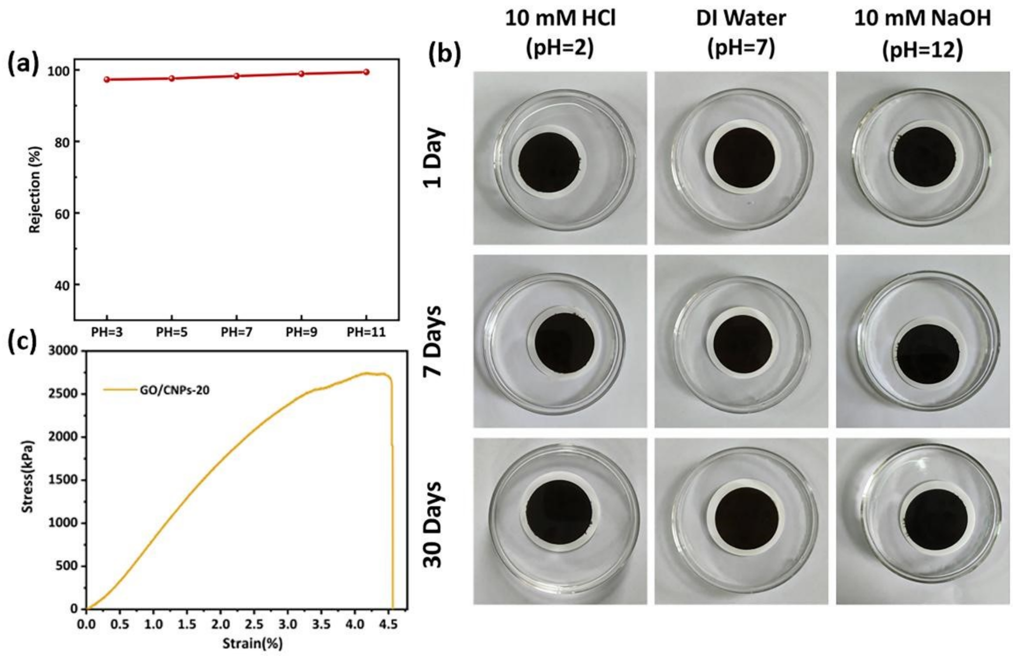

Fig. S14. (a) Rejection of MB(10ppm) at different pH of GO/CNPs-20 membrane; (b) Stability of GO/CNPs-20 membranes in 10 mM HCl, 10 mM NaOH, and DI water for 1 d, 7 d, and 30 d, respectively. PES filters were used as porous supports; (c) Stress-strain curve of GO/CNPs-20 membrane.

Table S2. Data of dyes for the molecule separation experiments.

Dyes	Abbreviation	pKa	Electrical charge	Mw (Da)	Molecular Stokes Radius (Å)
Methyl Orange	MO	3.4	-	327.33	4.44
Methylene Blue	MB	3.0	+	319.85	4.39
Rhodamine B	RhB	3.5	+	479.02	5.43
Bromocresol Green	BCG	4.68	-	698.05	6.62
Evans Blue	EB	1.5-2.5	-	960.81	7.82
